# Aerobic exercise regulates Rho/cofilin pathways to rescue synaptic loss in aged rats

**DOI:** 10.1371/journal.pone.0171491

**Published:** 2017-02-02

**Authors:** Yan Li, Li Zhao, Boya Gu, Jiajia Cai, Yuanyuan Lv, Laikang Yu

**Affiliations:** 1 Department of Exercise Physiology, Beijing Sport University, Beijing, China; 2 Key Laboratory of Physical Fitness and Exercise, Ministry of Education, Beijing Sport University, Beijing, China; Bilkent University, TURKEY

## Abstract

**Purpose:**

The role of exercise to prevent or reverse aging-induced cognitive decline has been widely reported. This neuroprotection is associated with changes in the synaptic structure plasticity. However, the mechanisms of exercise-induced synaptic plasticity in the aging brain are still unclear. Thus, the aim of the present study is to investigate the aging-related alterations of Rho-GTPase and the modulatory influences of exercise training.

**Methods:**

Young and old rats were used in this study. Old rats were subjected to different schedules of aerobic exercise (12 m/min, 60 min/d, 3d/w or 5d/w) or kept sedentary for 12 w. After 12 w of aerobic exercise, the synapse density in the cortex and hippocampus was detected with immunofluorescent staining using synaptophysin as a marker. The total protein levels of RhoA, Rac1, Cdc42 and cofilin in the cortex and hippocampus were detected with Western Blot. The activities of RhoA, Rac1 and Cdc42 were determined using a pull down assay.

**Results:**

We found that synapse loss occurred in aging rats. However, the change of expression and activity of RhoA, Rac1 and Cdc42 was different in the cortex and hippocampus. In the cortex, the expression and activity of Rac1 and Cdc42 was greatly increased with aging, whereas there were no changes in the expression and activity of RhoA. In the hippocampus, the expression and activity of Rac1 and Cdc42 was greatly decreased and there were no changes in the expression and activity of RhoA. As a major downstream substrate of the Rho GTPase family, the increased expression of cofilin was only observed in the cortex. High frequency exercise ameliorated all aging-related changes in the cortex and hippocampus.

**Conclusions:**

These data suggest that aerobic exercise reverses synapse loss in the cortex and hippocampus in aging rats, which might be related to the regulation of Rho GTPases.

## Introduction

As aging occurs and the mean life expectancy extends, the “normal” aging process is often associated with specific impairments in both learning and memory. A loss of synapses in neocortex and hippocampus appears to be the underlying cause of cognitive decline and memory deficit in the aged-brain [1]. Electron microscopy studies have also shown a substantial synapse loss in the prefrontal cortex of aged rhesus monkeys [2, 3]. The factors driving these alterations are still obscure. A feature of synapse loss is marked with a dramatic decrease of presynaptic boutons [4] and the expression of several synaptic proteins [5], synaptophysin in particular [6]. Microtubules and actin-containing microfilaments play complementary roles in regulating synaptic structure plasticity in neurons. Actin, the dynamics of which is tightly regulated by a variety of actin-binding proteins [7, 8], is particularly abundant in dendritic spines and mediates exploratory movements of growth cones as they respond to external cues [9]. Cofilin is one of the major actin depolymerization factor (actin-binding protein) in the brain and severs filamentous actin (F-actin) into short segments and creates free barbed ends for actin elongation [10], which plays a critical role in neurite growth and growth cone turning [2, 3, 11], receptor trafficking [12], and dendritic spinogenesis and synaptic plasticity [13, 14]. Misregulation of neuronal actin and cofilin is associated with a range of cognitive impairment and degenerative condition [15].

Rho-GTPase, a family of small G proteins among RhoA, Rac1, and Cdc42, play a pivotal role in synaptic morpho-functional changes relying on the dynamics of the F-actin-rich cytoskeleton present in spines. RhoA regulates actin polymerization and formation of actin-myosin filaments *via* p160ROCK, mDIA, LIMK, and MLC [16, 17]. Rac1 and Cdc42 stimulate F-actin polymerization through their common main downstream effector p21-activated kinase (PAK) which, acting on the LIM kinase (LIMK)-cofilin axes, stimulates actin polymerization [16, 17]. Rho-GTPases exert complementary functions to regulate actin dynamics and to direct complex neuronal functions [18, 19]. As an example, for RhoA to be active, there is a requirement for Rac1 inactivation [20]. Evidence *in vitro* identifies Rho-GTPase as key regulators of various steps in synaptic plasticity including neurite morphogenesis, motility, stability or collapse.

Exercise training has been instrumental in brain health and in neurodegenerative disorders. Exercise has been postulated to enhance brain function through various physiological mechanisms, including improved learning, memory and plasticity [21, 22], increased neuronal activation [23, 24] and enhanced neurogenesis [25]. Changes of neurogenesis in the adult brain [26] and of the expressions of genes related to synaptic plasticity [27], which are responsible for changing the number, structure and function of neurons [28], have also been observed. Even in very old animals a low level of adult hippocampal neurogenesis has been detected [26]. Also various exercise protocols have been used to study its beneficial effects, such as acrobatic exercise, wheel exercise, and treadmill exercise of low to high intensity, continuous or intermittent, and of short and long duration [27, 29]. However, depending on the intensity of training, exercise could be, instead of beneficial, deleterious to the brain, by causing increased free radicals, cytokine production and excitotoxicity [30]. Enriched environments have also been used, and all these modalities have the purpose of investigating specific exercise-induced plastic changes that occur in different brain regions [31]. Our recent study showed that in young healthy rats, aerobic exercise can increase the cortical synaptic density by regulating Rho family GTPases [32, 33]. Notably, most of the evidence is from young animals and Alzheimer’s disease-linked mutant animals. There is a shortage of exercise studies in aging animals addressing the role of different Rho-GTPase in regulating synaptic structural and proteins in the same way in different brain regions. Therefore, this study was designed to examine the aging-related alteration of Rho-GTPase and the modulatory influence of exercise training. This could be used to help understand the beneficial effects of exercise on aging-reduced brain function related to synaptic loss and how this could contribute to the development of protective strategies.

## Methods and materials

### Animals and exercise protocol

Young (2 months old) and old (24 months old) male Wistar rats (obtained from Vital River Laboratory Animal Technology Co. Ltd. Beijing, China) were used in the study and randomly assigned to young control (YC), old sedentary (O-SED), old low frequency exercise (O-LEX) and old high frequency exercise (O-HEX) groups. All rats were kept at a SPF (specific pathogen free) animal lab with free access to tap water and chow. The animal lab was controlled under a 12 h light: 12 h dark conditions (light time 07:00–19:00). The investigations were approved by the local ethics committee of Beijing Sport University and were carried out according to the requirements of The Guiding Principles for Care and Use of Animals. All animals were introduced to treadmill running for 5 d at 10 m/min, once a day for 10–20 min. Then, the O-LEX and O-HEX groups rats were subjected to a 12 w running protocol. In the 12 w training program, exercised animals ran at 12 m/min, 0 slope, for 60 min/d. The running frequency is 3 d/w for O-LEX, whereas 5 d/w for O-HEX. The animals were killed 2 d after the last exercise session to avoid the metabolic effects of the final run. Animal monitoring occurred daily, during which nutritional health, normal mobility and general health were checked. All animals maintained good health thoughout the experiment with no need for early euthanasia.

### Immunofluorescent staining

Rats were anesthetized with urethane, and the brain was fixed by transcardially perfused with 0.1 M phosphate-buffered saline (PBS) pH 7.4 followed by 4% paraformaldehyde in 0.1 M PBS. Then the brain was removed from skulls and postfixed at 4°C in 4% paraformaldehyde for 48 h, dehydrated with 30% sucrose in PBS at 4°C overnight, and frozen in liquid nitrogen coated with OCT. Coronal sections were made on a cryostat (CM1850, Leica, Wetalar, Germany) at 40 μm. Free-floating sections were washed in PBS three times for 10 min. Antigen retrieval was done by heating sections in sodium citrate buffer (10mM sodium citrate, 0.05% Tween 20, pH 6.0) at 95°C for 15 min. After washing in PBS, sections were permeabilized in 1% Triton X-100, washed, blocked with 10% BSA (0218054980, MP Biomidicals, Santa Ana, CA, USA) for 30 min at room temperature. After blocking, sections were incubated with primary antibody against synaptophysin (1:200, ab68851, Abcam, Cambrige, UK) at 4°C for 24 h. The next day, sections were rinsed three times at 10 min each in PBS, and then incubated in the secondary antibody: fluorescein (FITC)-conjugated affinipure goat anti-rabbit IgG (H+L) (1:200, SA00003-2, Proteintech Group, Chicago, IL, USA) for 60 min in the dark at room temperature. After washed, sections were mounted onto glass slides with antifade solution (070044-A, Cellchip Biotechnology, Beijing, China). The sections were visualized and taken photos using a fluorescence microscope (ECLIPSE 50i, Nikon, Melville, NY, USA). The fluorescence intensity of each section was quantified by ImageJ software (NIH).

### Protein extraction and western blot analysis

Immediately after animal sacrifice, the cortex and hippocampus were collected, frozen in liquid nitrogen quickly and stored in -80°C. Tissues were homogenized with RIPA buffer (R0278, Sigma-Aldrich, St. Louis, MO, USA) containing 50mM Tris-HCl pH 8.0, 150mM NaCl, 1% NP-40, 0.5% sodium deoxycholate, 0.1% SDS and protease inhibitors (04693159001, Roche, Mannheim, Germany). Lysates were then centrifuged at 13,000 *g* for 30 min at 4°C on the centrifuge (5424R, Eppendorf, Hamburg, Germany) and the supernatants were collected. Protein concentration was determined using the BCA method with BSA as a reference (23227, Pierce) on the xMark^™^ Microplate Absorbance Spectrophotometer (Bio-Rad Laboratories, Hercules, CA, USA). Samples were diluted in 2× sample buffer containing 50 mM Tris-HCl pH 6.8, 2% SDS, 10% glycerol, 0.1% bromophenol blue and 5% β-mercaptoethanol and then heated at 100°C for 15 min. 20 μg total protein was electrophoresed on 10–12% polyacrylamide gels (120V, 90–120 min) and electrophoretically transferred onto PDVF membrane (IPVH00010, Millipore, Billerica, MA, USA). Then the membrane was blocked with 5% BSA (0218054980, MP Biomidicals) in TBS containing 0.1% Tween 20 (0777, Amresco, Solon, Ohio) for 2 h at room temperature, incubated in fresh washing buffer for 10 min, and incubated with the following primary antibodies: RhoA (1:1500, ab68826, Abcam), Rac1 (1:1500, ab33186, Abcam), Cdc42 (1:1500, ab109553, Abcam), Cofilin (1:1000, ACFL02-A, Cytoskeleton, Denver, CO, USA) and GAPDH (1:4000, 60004-1-Ig, Proteintech Group) at 4°C overnight. After three washes in TBS-Tween 20, the membranes were incubated in horseradish peroxidase-conjugated anti-rabbit or anti-mouse second antibody (111-035-003 or 115-035-003, Jackson ImmunoResearch Laboratories, West Grove, PA, USA) for 1 h at room temperature. After repeatedly washed, the protein bands were visualized using Supersignal West Pico (34080, Pierce) and the signals were recorded on the ChemiDoc^™^ XRS+ System (Bio-Rad). The bands were quantified by Image Lab software (Bio-Rad), and normalized to GAPDH, which served as an internal control.

### Pull down assay

The activities of RhoA, Rac1 and Cdc42 from the cortex and hippocampus were measured using RhoA/Rac1/Cdc42 Activation Assay Combo Biochem Kit (BK030, Cytoskeleton). In brief, tissues were ground in liquid nitrogen and incubated in a lysis buffer 50 mM Tris-HCl pH 7.5, 0.5 M NaCl, 10 mM MgCl_2_, 2% Igepal and protease inhibitors with rocking at 4°C for 1 h. Sample lysates were centrifuged for 30 min at 16,000 *g* at 4°C and supernatants were collected into new tubes. The BCA method was used for quantitation of total protein concentration. 400 μg total protein was incubated with 50 μg rhotekin-RBD beads (for RhoA activation assay) or 10 μg PAK-PBD beads (for Rac1/Cdc42 activation assay) on a rotator at 4°C for 1 h. Then the beads were pelleted by centrifugation at 5,000 g for 1 min at 4°C and the supernatant was removed. After washing with a wash buffer, the beads were resuspended and centrifuged to remove the supernatant. The bound proteins were suspended in 20 μl 2× Laemmli sample buffer and boiled for 2 min. The sample was separated in 15% SDS-PAGE for the western blot analysis with the following primary antibodies: RhoA(1:1500, ab68826, Abcam), Rac1(1:1500,ab33186, Abcam), Cda42(1:1500, ab109553, Abcam).

### Statistical analysis

Results are expressed as mean ± SEM, and n denotes the number of animals used in each experiment. Statistical analysis was conducted using one-way analysis of variance followed by Tukey post hoc test for multiple comparisons. A value of p < 0.05 was considered significant.

## Results

### RhoA

**Fig 1:**
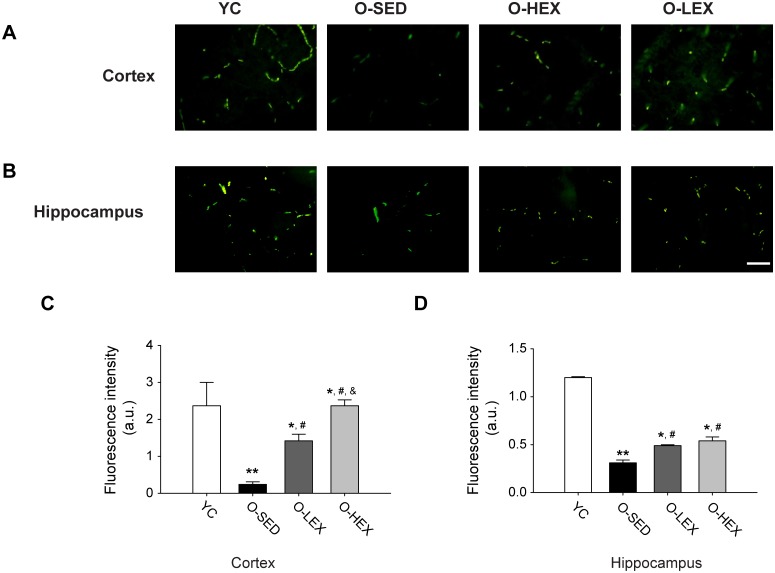
Exercise reverses the synapse loss during aging. **(A-B)** Representative positive immunofluorescence staining of synaptophysin in the cortex (A) and hippocampus (B) in four groups. **(C-D)** Summary of the mean fluorescence density in the cortex (C) and hippocampus (D) in four groups. Values are mean and standard error of the mean, n = 5 in each group. ** P< 0.01 vs. YC, ^#^P< 0.05 vs. O-SED, ^&^ P< 0.05 vs. O-LEX. Bar = 100 μm.

**Fig 2:**
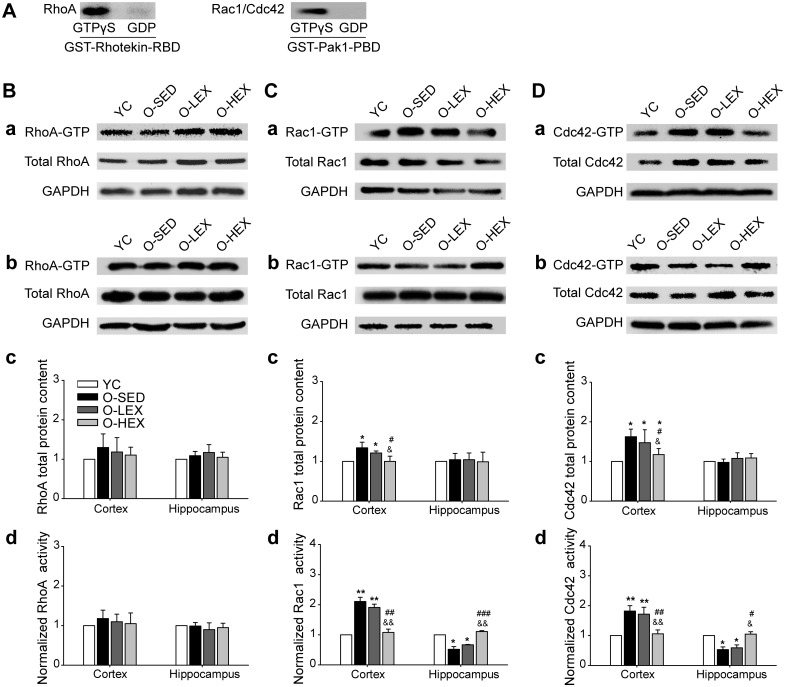
Exercise regulated the expressions and activities of Rho GTPases in the cortex and hippocampus in aging rats. **(A)** Positive and negative controls of Rho GTPasee in pull down assays. **(B)** Effects of exercise on the expressions and activities of RhoA. **(a-b)** Immunoreactive bands corresponding to RhoA active and total protein and GAPDH in the cortex (a) and the hippocampus (b). **(c-d)** Summarized data of RhoA total protein levels (c, as a ratio to GAPDH) and activities (d, as a ratio to total protein) in the cortex and the hippocampus. n = 5 in each group. **(C)** Effects of exercise on the expressions and activities of Rac1. **(a-b)** Immunoreactive bands corresponding to Rac1 active and total protein and GAPDH in the cortex (a) and the hippocampus (b). **(c-d)** Summarized data of Rac1 total protein levels (c, as a ratio to GAPDH) and activities (d, as a ratio to total protein) in the cortex and the hippocampus. n = 5 in each group. **(D)** Effects of exercise on the expressions and activities of Cdc42. **(a-b)** Immunoreactive bands corresponding to Cdc42 active and total protein and GAPDH in the cortex (a) and the hippocampus (b). **(c-d)** Summarized data of Cdc42 total protein levels (c, as a ratio to GAPDH) and activities (d, as a ratio to total protein) in the cortex and the hippocampus. Values are mean and standard error of the mean, n = 5 in each group. *P< 0.05 vs YC, **P< 0.01 vs YC, ^#^P< 0.05 vs O-SED, ^##^P< 0.01 vs. O-SED, ^###^ P< 0.001 vs. O-SED, ^&^P< 0.05 vs. O-LEX, ^&&^P < 0.01 vs. O-LEX.

Synaptophysin, a marker of excitatory presynapses, is primarily present within vesicles, and its expression level can be used to examine synapse loss [34]. As shown in Fig 1 (immunofluorescence with synaptophysin), the labeling of synaptophysin, showed a dramatic decline in the cortex and hippocampus of aged rats, including O-SED, O-LEX and O-HEX, especially in O-SED (p < 0.01). RhoA, often in colocalization with synaptophysin, is widely distributed throughout the cortex and hippocampus, it is present in axon terminals (especially excitatory synaptic terminals), and in dendrites, where it appears associated to microtubules [35]. Rho-GTPase content occurs in membrane (active) and cytosolic (inactive) pools. Western blot and pull-down assay were performed to detrermine whether changes in synaptophysin were concomitant to the alteration in expression and/or activity of RhoA. In total expression and activity, RhoA showed a slight but not statistically significant increase in aged-brains compared to that in Young (Fig 2). The lack of change in expression or activity was unexpected in view of the robust decrease in synaptophysin revealed by the immunofluorescence. It is possible that only distribution of RhoA was to alter in aged brains, rather than to change in net expression or activity.

**Fig 3:**
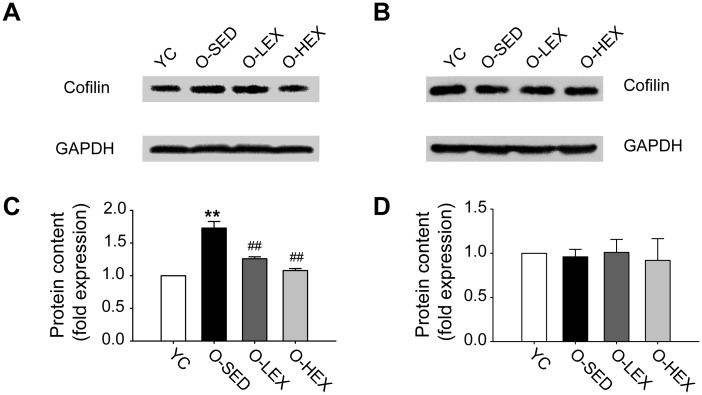
Exercise decreased the increased expression of cofilin in the cortex during aging. **(A-B)** Immunoreactive bands corresponding to cofilin and GAPDH in the cortex (A) and hippocampus (B). **(C-D)** Summarized data of cofilin protein levels (as a ratio to GAPDH) in the cortex (C) and hippocampus (D). Values are mean and standard error of the mean, n = 5 in each group. ** P< 0.01 vs. YC, ^##^ P< 0.01 vs. O-SED.

Cofilin is the major downstream substrate of the Rho GTPase family and plays a critical role in neurite growth [11, 36]. Also we examined the cofilin contents, which protein expression values described for Western blotting are relative optical densities (protein bands/GADPH bands). The concentration of cofilin in the cortex and in the hippocampus showed difference in aging brains compared with that of Young brains (Fig 3). There was an increase of cofilin in aged cortex, and no changes were observed in the hippocampus.

Based on several observations of the positive effect of different exercise protocols on synaptic and structural proteins, we analyzed the effects of aerobic exercise on synaptic loss in aged brains. After exercise training, synaptophysin was significantly augmented in O-LEX and O-HEX compared with that in O-SED though it was still lower than that in Young (p < 0.05) (Fig 1). And as shown in Fig 1, in the cortex the effect of exercise appeared to salient better in O-HEX. With increased synaptophysin, exercise training induced a decrease of cofilin in both O-LEX or O-HEX in cortex (p < 0.01) (Fig 3), and that was very similar to what was observed in synaptophysin, the value of O-HEX was lower than that in O-LEX. But there was no change in total Rho A expression, a small but not statistically significant decrease in O-HEX. Also aerobic exercise did not affect the activity of RhoA in the cortex and hippocampus.

These results indicated that during aging the synaptophysin has been shown to be reduced in the cortex and the feature was accompanied by cofilin aggregation that were associated with synaptic loss, while in the hippocampus, the synaptic loss was not the result of cofilin aggregation. Aerobic exercise training partly but significantly prevents this event. And these results are consistent with our previous findings [32] exercise increased the synaptic density in adult rats.

### Rac1

Rac1 immunohistochemistry has shown that Rac1 is ubiquitously distributed in the brain neuropil and does not specifically concentrate in synapses [35]. In this study Rac1 protein analysis of the cortex and the hippocampus was shown in Fig 2 (and comparison of the young control). The changes of Rac1 were different in cortex and in hippocampus. The total expression of Rac1, showed a significantly increased in cortex of O-SED, and no remarkable alteration was detected in hippocampus. As to the activity of Rac1, there was in cortex an obviously increase, whereas in hippocampus a markedly decrease, suggesting some activity of this small G protein in senile cortex and some inactivity in senile hippocampus. In hippocampus this decrease was not reflected in the total contents was probably duo to the fact that cytosol pools contribute mainly to total Rac1, and with aging the changes primarily emerged in membrane contents (binding with GTPase). This result suggested that with aging the change of Rac1 appears to diverge in different brain regions, an increase in total expression and activity were observed in cortex, while no change in expression and a decrease in activity were found in hippocampus.

After exercise training for 12 w, in the cortex, the Rac1 total expression and activity in O-HEX were significantly decreased even to approximate that in Young. A slight but not statistically significant decrease in total expression and an increase in activity was observed in O-LEX. In the hippocampus, O-LEX training had no effect on the Rac1 in expression or activity and O-HEX training caused only a marked increase in Rac1 activity and no change in Rac1 expression, compared with that in O-SED.

### Cdc42

Cdc42 was revealed by western blot analysis to indicate that the aging cortex shows an increase of total expression and activity, while in the hippocampus no significant changes were observed in total expression, and there was a significant decrease in activity (Fig 2). These data point out that Cdc42 changes diverge in aging the cortex and the hippocampus. Aerobic exercise resulted in a decrease in the total amount of Cdc42 and its activity in the cortex, also increased activity and no effect on Cdc42 protein in the hippocampus (Fig 2).

## Discussion

This study is the first to show that some important insights concerning the molecular mechanism underlying aerobic exercise training in age-related brain structure changes. First, we analyzed the changes of synaptophysin and cofilin with aging. The results show that the levels of synaptophysin decreased and cofilin aggregated in the cortex of aged rats. Compared with O-SED group, the animals that performed 12-week aerobic exercise had higher synaptophysin levels but lower cofilin. As we know, several studies have examined the effect of different protocols of exercise on synaptic proteins in the rat brain, but we find little material in the literature that examines the effects on aging animals. Synaptophysin is a 38-kd glycoprotein localized in synaptic vesicle membranes. The main functions of synaptophysin include docking, fusion, and endocytosis, otherwise known as membrane trafficking [37]. It is considered to be involved in synaptic loss and plasticity. Cofilin is an actin-binding protein and its aggregates are associated with synaptic loss in neurodegeneration and brain aging. Our data indicate that cofilin changes could be related to adjustments of synaptic protein traffic induced by training in aged animals, whereas synaptophysin changes could reflect neuronal remodeling which may also be related to synaptic plasticity and efficiency. Also our present results suggest that hippocampal synapse loss may be another pathway because cofilin showed no aggregation in the aging hippocampus.

Although studies in different models from adult rodents animals have shown that exercise can have a beneficial effect on neuroplasticity by improving neurogenesis, brain structure and function, little material focuses on exercise protocol. As individuals age, cardiorespiratory fitness decreases and exercise capacity declines. In this study we choose two frequencies of forced exercise (3 times per week and 5 times per week) at the same treadmill intensity [38], could have different effects on the aging brain. The results show that old rats (24 month) can finish the moderately intense (12 m/min, 60 min/d) exercise. In addition to exercise, our protocol also contain stress. Exercise is often associated with higher levels of circulating corticoid hormones. We started the treadmill running with a 5 d adaptation period at a low level of moderate intensity, then followed with moderately intense running for 12 w. We have found that the corticosterone levels were increased at 30 min after exercise and returned to baseline during a resting state (unpublished materials). We chose to test longer periods of exercise based on evidence showing that for improving cognitive function, intervention via shorter period exercise programs (less than 12 weeks) failed to improve cognitive performance (other than the short-lived benefits to verbal fluency [39]). Moreover, exercise programs with a duration of less than 12 weeks that are not at least of moderate intense (>60% VO2max) may not produce significant enhancement in cardiorespiratory fitness [40, 41]. While enhancement in cardiorespiratory fitness can increase cerebral blood flow and improve oxygen utilization. Berchtold [42] have suggested that the effects of exercise may depend on factors such as duration of exercise exposure, type of exercise performed and possibly other, still uncharacterized variables. Therefore, our protocol followed moderate-intensity exercise and longer periods of exercise.

Because cofilin aggregation is associated with synaptic loss in aging brain and Rho-GTPase regulates synaptic plasticity, we sought to determine whether Rho-GTPase were altered in aging brain and exercise had benefical effects on them. Increased cofilin level in neurons reaches a threshold, neuronal functions and synaptic connection will be impaired. It is reasonable to deduce that a balanced activity among cofilin-regulatory proteins may reverse aging-associated adverse structural and functional remodeling of synapse during brain aging. Here we analyzed the changes of Rho-GTPase, cofilin upstream regulator, namely RhoA, Rac1 and Cdc42 at aged rats. There are two main findings in this section study. One is no major changes for RhoA level or activity, and that there is an increased expression or activity for Rac1 and Cdc42 in aged rat cortex, suggesting that normal aging physiology affects Rac1 and Cdc42 more than RhoA. The second finding is the altered feature of Rac1 and Cdc42 in the cortex and hippocampus is different. In the hippocampus, no changes for Rac1 or Cdc42 expression and decreased activation were observed. Expression of a constitutively active form of Rac1 and Cdc42 would be expected to enhance the activity of Pak1 and through LIMK1 cause cofilin phosphorylation (inactivation) and decreased cofilin activity results in subsequent local expansion of actin filament structures and synaptic stabilization [43]. The results indicate that during brain aging the cortex is more vulnerable to aging than the hippocampus in rodents [44–46], and Rho-GTPase levels in the two brain regions show aging-related divergence. These outcomes are in agreement with previous studies demonstrating that hippocampal synapse number seems to be preserved in aged rodents in some regions relative to young rats [47–48].

Our findings also point to an adjustment of the central nervous system by the functional reorganization of the Rho GTPase pathways after exercise, possibly involving synaptic and neuronal remodeling processes mediated by up- and down-regulation of protein synthesis, degradation and trafficking. The data show that on receiving aerobic exercise training Rac1 or Cdc42 total expression and activity are decreased in the cortex, also Rac1 or Cdc42 activity are increased in the hippocampus. After the same intensity of exercise protocols, the effects of HEX group were more sensitive than LEX group, suggesting that exercise must be of a certain intensity to have effect. This is consistent with the toxicological effects reported in our previous study [49] and for an individual exercising at an intensity level suitable for the individual, more exercise above this level has a greater effect on the positive stimulation in the body.

## Conclusions

The results here indicate that the aging animals in this study did have the common aging features such as synaptic loss with a decreased synaptic protein and cofilin aggregation. After 12-weeks of aerobic exercise, all of these changes were diminished, which indicated the efficacy of the training program slowing down the progress of age-related changes and suggests that exercise may be a non-drug therapeutic way to reduce the risk of dementia independently of the diet. But clearly exercise inducing multiple molecular pathways needs to be a major focus going forward.
